# A real‐world expert opinion on the use of a combination of hyaluronic acid and amino acids in the management of acute and chronic wounds

**DOI:** 10.1111/iwj.14671

**Published:** 2024-02-23

**Authors:** Keith Harding, Douglas Queen

**Affiliations:** ^1^ Cardiff University Wales UK; ^2^ Medicalhelplines.com Inc Toronto Ontario Canada

The hierarchy of clinical evidence in support of medical devices ranges from case studies to randomised controlled trials (RCTs), systematic reviews and meta‐analyses. While RCTs are seen as the gold standard for medicinal products, case studies provide some real‐world insight as to the usefulness of medical devices, especially within the management of wounds.

As wound carers/healers, we understand the complexity of these patients, with a variety of wound types, co‐morbidities and a range of potential clinical outcomes. In wound patients however, like many other clinical conditions, patients are individual with their own unique challenges and outcomes. This is where clinical case studies can provide insight into the intricacies of the individual rather than the collective outcomes of a pooled population in a RCT: a real‐world, personalised view. This could be a useful stage in supporting interventions and supplement the data derived from RCTs.

In the two articles within this supplement, the authors present a review of the previously published clinical support for a unique hyaluronic acid and amino acids combination product (Vulnamin®). It also presents additional clinical evidence obtained through a consensus meeting of an international group of experts regarding their clinical experience with the use of the combination product in difficult‐to‐treat wounds. Their objective was to reach a consensus on how and when to utilise such products to provide a cost‐effective, convenient option in all healthcare settings to improve outcomes for patients.

Members of the Expert Panel presented their clinical experience with the product range. The case reports presented were obtained in Italy, Poland, Turkey and the United Kingdom, with data collection in accordance with the principles of Good Clinical Practice. From this real‐world information, the panel of experts presented recommendations for the use of the product range in clinical practice. The 14 case studies presented demonstrate successful use of this hyaluronic acid and amino acids combination to be effective in the management of acute and chronic wounds.

While the experts provide guidance on the use of such an approach in a variety of clinical situations, they do recommend that future, large‐scale, randomised, controlled clinical trials are required in different clinical settings. In addition, the experts recommend further health economic data and reimbursement studies are carried out.

The information provided gives a real‐world picture on the flexibility of this combination product in the management of difficult to heal wounds, while recognising the need for further data. From this case series and supportive clinical review, the expert clinicians recommend considering the use of a combination product of hyaluronic acids and amino acids in several clinical situations.

## CONFLICT OF INTEREST STATEMENT

No interest is declared by both authors in the sponsor of the articles in this supplement.

